# Effect of increasing fruit and vegetable intake by dietary intervention on nutritional biomarkers and attitudes to dietary change: a randomised trial

**DOI:** 10.1007/s00394-017-1469-0

**Published:** 2017-05-30

**Authors:** Susan J. Duthie, Garry G. Duthie, Wendy R. Russell, Janet A. M. Kyle, Jennie I. Macdiarmid, Vanessa Rungapamestry, Sylvia Stephen, Cristina Megias-Baeza, Joanna J. Kaniewska, Lindsey Shaw, Lesley Milne, David Bremner, Karen Ross, Philip Morrice, Lynn P. Pirie, Graham Horgan, Charles S. Bestwick

**Affiliations:** 1 Natural Products Group, Division of Lifelong Health, Rowett Institute of Nutrition and Health, University of Aberdeen, Aberdeen, UK; 2 Public Health Nutrition Research Group, Rowett Institute of Nutrition and Health, University of Aberdeen, Aberdeen, UK; 3 Biomathematics and Statistics Scotland, Aberdeen, UK; 4 School of Pharmacy and Life Sciences, Robert Gordon University, Garthdee Road, Aberdeen, AB10 7GJ UK

**Keywords:** Fruit, Vegetables, Human intervention, Dietary change, Biomarkers, Attitudes

## Abstract

**Purpose:**

Low fruit and vegetable consumption is linked with an increased risk of death from vascular disease and cancer. The benefit of eating fruits and vegetables is attributed in part to antioxidants, vitamins and phytochemicals. Whether increasing intake impacts on markers of disease remains to be established. This study investigates whether increasing daily intake of fruits, vegetables and juices from low (approx. 3 portions), to high intakes (approx. 8 portions) impacts on nutritional and clinical biomarkers. Barriers to achieving the recommended fruit and vegetable intakes are also investigated.

**Method:**

In a randomised clinical trial, the participants [19 men and 26 women (39–58 years)] with low reported fruit, juice and vegetable intake (<3 portions/day) were randomised to consume either their usual diet or a diet supplemented with an additional 480 g of fruit and vegetables and fruit juice (300 ml) daily for 12 weeks. Nutritional biomarkers (vitamin C, carotenoids, B vitamins), antioxidant capacity and genomic stability were measured pre-intervention, at 4-, 8- and 12 weeks throughout the intervention. Samples were also taken post-intervention after a 6-week washout period. Glucose, homocysteine, lipids, blood pressure, weight and arterial stiffness were also measured. Intake of fruit, fruit juice and vegetables was reassessed 12 months after conducting the study and a questionnaire was developed to identify barriers to healthy eating.

**Results:**

Intake increased significantly in the intervention group compared to controls, achieving 8.4 portions/day after 12 weeks. Plasma vitamin C (35%), folate (15%) and certain carotenoids [α-carotene (50%) and β-carotene (70%) and lutein/zeaxanthin (70%)] were significantly increased (*P* < 0.05) in the intervention group. There were no significant changes in antioxidant capacity, DNA damage and markers of vascular health. Barriers to achieving recommended intakes of fruits and vegetables measured 12 months after the intervention period were amount, inconvenience and cost.

**Conclusion:**

While increasing fruit, juice and vegetable consumption increases circulating level of beneficial nutrients in healthy subjects, a 12-week intervention was not associated with effects on antioxidant status or lymphocyte DNA damage.

**Trial registration:**

This trial was registered at Controlled-Trials.com; registration ISRCTN71368072.

**Electronic supplementary material:**

The online version of this article (doi:10.1007/s00394-017-1469-0) contains supplementary material, which is available to authorized users.

## Introduction

Low consumption of plant-based foods, including fruits and vegetables, is associated with an increased risk of several human chronic non-communicable diseases including hypertension, cardiovascular disease (CVD), stroke, obesity, diabetes, osteoporosis and certain cancers and with high all-cause mortality [1–5]. In 2009 it was estimated that in excess of 2 million deaths and 26 million disability-adjusted-life-years (DALYs; 1.8%) could be attributable to suboptimal fruit and vegetable consumption worldwide [6]. Recent data suggest that these figures may be considerably higher, with nearly 8 million premature deaths attributable to a fruit and vegetable intake below 800 g per day [5]. Modelling data suggest that 31% of ischemic heart disease, 19% stroke, 20% oesophageal cancer, 19% gastric cancer and 12% lung cancer cases globally could be avoided by increasing the daily intake of fruits and vegetables to at least 400 g per day [7], while 15 000 deaths each year could be avoided if similar dietary guidelines were followed in the UK [8]. In the recent UK National Diet and Nutrition Survey (NDNS), 70% of all men and women sampled reported eating less than the recommended minimum 5 daily portions (400 g), with 62% of both sexes consuming fewer than 3 portions of fruits and vegetables each day [9].

Low fruit and vegetables consumption is not confined to high-income countries but is prevalent across many nations. In a recent study, 77.6% of men and 78.4% of women sampled from 52 low- and middle-income countries reported consuming less than 400 g of fruit and vegetables per day, the minimum recommended by the World Health Organisation (WHO) panel on diet, nutrition and prevention of chronic disease [6].

While early observational and case-controlled human studies provided evidence of a substantial protective effect of fruit and vegetable consumption on human chronic disease risk [10–12], large-scale prospective and intervention studies, together with several recent meta-analyses, have reported weaker associations [1, 3, 13, 14]. However, increased fruit and vegetable intake remains consistently associated with a reduced incidence of CVD, stroke and diabetes [5, 15–19] and with a lower rate of death from all causes and from certain cancers [1, 3, 5]. Moreover, high fruit and vegetable consumption is linked with changes in specific antioxidant markers or early disease indicators associated with risk, including cholesterol oxidation products, plasma antioxidant capacity, oxidised DNA base damage and total circulating glucose, homocysteine, lipids, blood pressure (BP) and body weight [18, 20–24].

Fruits and vegetables are rich sources of a wide range of beneficial nutrients and non-nutrients including fibre, vitamins (particularly A, B and C), minerals (selenium and potassium), antioxidants (carotenoids and tocopherols) and phytochemicals including flavonoids, glucosinolates and isothiocyanates [1, 4, 12]. Mechanistically, antioxidant compounds and vitamins could reduce the risk of cancer and vascular disease by scavenging reactive oxygen species (ROS) and other free radicals and preventing tissue DNA and lipid oxidation in arteries [25–27]. Other potential mechanisms attributed to antioxidants and B vitamins present in fruits and vegetables include maintaining endogenous DNA stability, lowering total plasma homocysteine (a vascular toxin) and maintaining blood pressure (BP) and endothelial cell function and health [26–28].

The aims of this multidisciplinary study were (1) to investigate whether increasing the daily intake of fruit and vegetables (480 g) and fruit juice (300 ml), would improve the circulating level of vitamin C and other antioxidant nutrients in a group of habitually low consumers; (2) to establish whether increasing fruit and vegetable intake accordingly impacted positively on anti-oxidative capacity in plasma and cellular antioxidant enzyme activity; (3) to establish whether providing additional fruits, vegetables and juices altered total energy intake and intake of other key macro- and micronutrients; and (4) to identify the perceived barriers to increasing fruit and vegetable consumption 12 months after the intervention. Particular emphasis was placed on increasing intake through provision of taxonomically diverse and readily available (in UK) variety of fruits and vegetables, representing an intake regime compatible with sustained consumption. We believe that this is the first study to assess the impact of increasing fruit and vegetable consumption on nutrient status, antioxidant biomarkers of health and long-term attitudes to increasing consumption within the context of a “real world” setting.

## Materials and methods

### Participants

Participants from the NE of Scotland were recruited through the Human Nutrition Unit (HNU) at the Rowett Institute of Nutrition and Health (RINH), the University of Aberdeen. Participants were recruited through the RINH recruitment database and by advertising the study in the local media. Inclusion criteria were for healthy men and women with a habitually low fruit and vegetable intake, non-smoking, non-medicating, with self-reported normal bowel function, aged 38–60 years and with a BMI between 18 and 39 kg/m^2^. Participants were excluded if they reported consuming more than three portions of fruit and vegetables per day as assessed by a food frequency questionnaire (The Scottish Collaborative Group FFQ version 6.6; http://www.foodfrequency.org), smoked, had a chronic medical condition (including inflammatory bowel disease), had a known allergy to plant products and/or took medication (including contraceptive tablets, hormone replacement therapy or thyroid drugs). This study was conducted according to the guidelines laid down in the Declaration of Helsinki and all procedures involving human subjects was approved by the Ethics Committee of the Rowett Institute of Nutrition and Health, University of Aberdeen (study code: 09/003) following review by the North East of Scotland Research Ethics Committee. Written informed consent was obtained for all subjects. Participants were informed by way of an information sheet as to the purpose and risks of the study. This trial is registered at Controlled-Trials.com; registration ISRCTN71368072.

### Study design

**Fig. 1:**
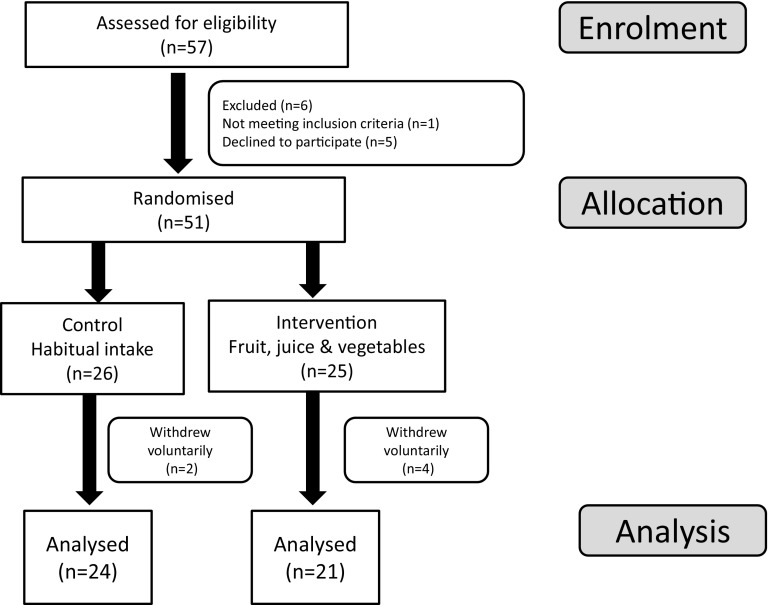
Trial profile

Fifty-seven volunteers were assessed for entry into the study (Fig. 1). Six of these did not meet the inclusion criteria or declined to participate further. A total of fifty-one participants were randomly assigned (by sex, age and BMI) by a statistician to the study groups (*n* = 26 in the control group and *n* = 25 in the treatment group). Participants were paired with others of a similar sex, age and BMI, and treatment assigned randomly within each pair. It was not possible to use an optimal pairing across all volunteers, as it was necessary to start some participants onto study before all had enrolled across both phases. Participants in the control group were asked to maintain their habitual diet that included 3 or fewer portions of fruits and vegetables each day. Participants in the intervention group were asked to consume approx. 480 g of commonly available and taxonomically diverse fruits, vegetables and fruit juices (max. 300 ml per day) in addition to their normal intake. All fruit, vegetables and juice were provided (twice weekly) to the participants. The same fruits and vegetables were provided to all volunteers unless a participant declared a strong dislike for a particular item, in which case it was replaced. Participants were instructed to eat all of the foods provided, ensuring equivalent consumption of fruits and vegetables. Advice on preparation, storing and cooking methods was provided at the start of the intervention and throughout the study. Written instructions on preparation, including options on common preparation practices, was supported by twice-weekly personal contact with participants during fruit and vegetable collection. In addition, all participants were offered telephone or e-mail support throughout the study. The fruit and vegetables included apples, oranges, soft fruit and berries, broccoli, Brussels sprouts, cabbage, carrots, cauliflower, celery, cucumber, mixed lettuce, spinach, sweet pepper and tomatoes. Participants were supplied with a record sheet to record what fruit and vegetables were eaten daily, and if they were unable to eat all the foodstuffs each day. The study was carried out over a 20-week period and included a 2-week run-in period where participants consumed their habitual diet, a 12-week intervention and a 6-week washout period where fruit and vegetable provision was withdrawn and participants were allowed to eat as they wished. A washout time point was included to determine the efficacy of the nutritional and functional biomarkers of fruit and vegetable intake.

Blood sampling was carried out on six occasions; pre-run-in (time −2), baseline (time 0), intervention (weeks 4, 8 and 12) and post-washout (week 18). The study was carried out between March and August over a period of 2 years (2011 and 2012) to control, as much as possible, for seasonal variation. Forty-five participants completed the study (Fig. 1).

### Dietary intake assessment and compliance

Dietary intake (energy, micro- and macronutrient) and compliance was assessed using 4-day weighed food diaries before (pre-run-in), during (weeks 4 and 12) and after (post-washout) the intervention. Diaries were analysed using WinDiets 2005. Compliance during the intervention was assessed by calculating the total number of portions of fruit and vegetables each participant reported consuming over the 4 days for each of the time points. Portion sizes were taken from the National Health Service (NHS) “Livewell” website [29], which states that an adult portion is 80 g for fruit and vegetables, 30 g for dried fruit and 150 ml of fruit and/or vegetable juice. To calculate the number of portions, the weight of each fruit, vegetable and fruit juice serving recorded was divided by the adult portion size for that item. Using this method, the total number of portions reported as consumed for each 4-day food diary was calculated and divided by 4 to give the average number of portions consumed per day. As 150 ml of fruit or vegetable juice counts only as a maximum of one portion per day, any juice consumed above this amount was recorded as a proportion of an additional portion. Compliance to the intervention was further assessed by participants recording all foodstuffs not consumed on a weekly checklist of all items provided.

### Sampling and biomarker analysis

At each visit, fasted blood (12 h) was collected from the antecubital vein into vacutainers containing EDTA as anticoagulant. EDTA-treated whole blood was incubated with ascorbic acid (1% w/v) for 1.5 h in the dark, snap frozen in liquid nitrogen and stored at −80 °C for folate analysis. All remaining EDTA blood was centrifuged at 2400×*g* for 15 min at 4 °C and the plasma aliquoted, snap frozen and stored as above. Lymphocytes were isolated from the buffy coat by density gradient centrifugation [30].

Plasma and whole-blood folate and plasma B12 were measured by radioassay [31]. Plasma homocysteine was measured by gas chromatography [31]. Plasma vitamins B2, C, carotenoids (α- and β-carotene, β-cryptoxanthin, lycopene, lutein/zeaxanthin) and tocopherols (α- and γ) were measured by HPLC [32, 33]. Plasma total cholesterol, low density lipoprotein (LDL) cholesterol, high density lipoprotein (HDL) cholesterol, triglycerides (TG) and glucose were measured using a Konelab 20 Clinical Chemistry Analyser (Thermo Scientific, Passau, Germany). Plasma antioxidant capacity was measured using the trolox equivalent antioxidant capacity (TEAC), hydroxyl radical antioxidant capacity (HORAC), ferric reducing antioxidant power (FRAP) and total phenol assays in EDTA-treated plasma spectrophotometrically [34–37]. Antioxidant enzyme activity in EDTA-treated red blood cell membranes [glutathione peroxidase, catalase, superoxide dismutase (SOD) and glutathione reductase] were measured as described previously [38–41]. Endogenous DNA strand breakage was measured in lymphocytes isolated from EDTA-treated whole blood using single cell gel electrophoresis [30]. Ability to resist an oxidative stress was measured in lymphocytes exposed to hydrogen peroxide (H_2_O_2_; 200 μm for 5 min on ice) before analyses of induced DNA strand breakage [30]. All analyses were carried out on blinded samples.

### Blood pressure and arterial stiffness

Blood pressure (on average three consecutive readings) was measured at each visit to the HNU with a sphygmomanometer (OMRON705CP) according to guidelines from the British Hypertension Society. Arterial stiffness was assessed by pulse contour analysis (Pulse Trace PCA, Micromedical Ltd).

### Attitudes towards increasing and sustaining fruit and vegetable intake

Attitudes towards consuming fruit and vegetables were explored using a questionnaire designed for the study. The questionnaire included both open and closed questions about barriers to healthy eating, including cost, taste, effort of preparation and cooking, availability, and family acceptance that may inhibit fruit and vegetable intake [42, 43].

The questionnaire was pre-tested for interpretation and ease of completion using cognitive interviewing techniques and modified accordingly. All participants were contacted 10–12 months after the intervention and sent the questionnaire by post to complete. A reminder letter and second questionnaire were sent to non-responders 2 weeks after the initial mailing.

### Statistical analyses

Study sample size was determined for the primary outcome of plasma vitamin C. Based upon a previous human study [44], an SD of 30% was used for calculating sample size. An expected decrease/increase of 30% in vitamin C, with an SD of 30%, power of 80% and a significance level of 5%, yields the need for 20 participants in each group to detect significant differences between treatments. Calculations were based upon tests comparing 2 means with 2-sided equality.

For descriptive data, results are generally presented as mean ± SEM unless otherwise stated. All participants who completed the study were included in the statistical analysis which was carried out using GenStat.v13 (VSN International, UK). Blood biomarkers at each time point were assessed on normal or log-transformed data (depending on distribution) using 2-way analysis of variance (ANOVA), with terms for treatment and sex and their interaction, and with baseline values (time 0), BMI and age as covariates. *P* < 0.05 was considered statistically significant. We also carried out a mixed model analysis of the data at all time points combined, with participant and week within participant as random effects, and treatment, week, sex and their interactions as fixed effects, and baseline values as an additional fixed effect. As linear trends over time were not expected, week was considered as an ordered categorical factor rather than a numerical covariate. A first-order autoregression correlation structure was assumed for time points. Analysis was carried out using the REML directive in GenStat.v13 (as above). These mixed model analyses confirmed the results of the ANOVAs at each time point, and so the results presented are based upon the latter, for clarity and to avoid the extra assumptions required in the joint analysis.

To test for differences in dietary intake and mean number of portions of fruit and vegetables reported as consumed between treatment groups with time (compliance), independent sample t-tests were conducted at baseline (time 0), throughout the intervention (weeks 4 and 12) and post-washout (week 18). For analysis of data collected by questionnaire (sustaining fruit and vegetable intake), for questions using a 5-point Likert scale, the points were collapsed to 3 for comparison (agree, neither agree nor disagree, disagree), collated and descriptively analysed, giving frequencies and percentages applied to the quantitative questions. The responses to the open-ended questions were grouped by 2 researchers independently and results compared [45].

## Results

### Recruitment

Fifty-seven people were screened prior to the intervention. One did not meet the inclusion criteria (low BMI) and five declined to take part in the study (undisclosed reasons). The remaining 51 participants were stratified by sex, BMI and age and randomised to two groups, control or intervention. Of these, three withdrew before week 2, two withdrew before week 3 and one withdrew before week 4. Forty-five participants completed the study, with 21 (9 men, 12 women) in the intervention group and 24 (10 men and 14 women) in the control group (Fig. 1).

### Participant characteristics

The age of the participants ranged from 39 to 58 years with a mean age of 48 years. Mean BMI was 26.48 ± 3.66 kg/m^2^ with no underweight participants, 31% normal weight, 56% overweight and 13% obese participants. More women than men were recruited (26 versus 19) and the number of participants that completed the study in each group was slightly unbalanced (24 control participants versus 21 in the intervention group). There was no significant difference between the groups with regard to age and BMI at the start of the study (Table 1) and no difference in calculated macro- and micronutrient intake (Table 2). Similarly, the vast majority of plasma and whole-blood nutrients and functional biomarkers were similar between the two treatment groups (Tables 3, 4, 5). However, participants in the intervention group had significantly lower plasma γ-tocopherol and HORAC levels at time 0, compared with subjects in the control group.

**Table 1:** Participant characteristics at baseline by intervention group

	Control (*n* = 24)	Intervention (*n* = 21)
Age (y)	48.5 ± 4.8 (39–57)	48.3 ± 5.6 (41–58)
Men (*n*)	10	9
Women (*n*)	14	12
BMI	26.0 ± 3.4 (18.9–31.1)	26.6 ± 3.9 (20.6–35.8)

Values are mean ± SD, (minimum–maximum)

**Table 2:** Dietary intake by intervention group

Intake	Group	Pre-run-in	Week 12	Post-washout
Energy (kJ)	I	8799 (2437)	8010 (3267)	7619 (2539)
	C	8610 (2690)	8259 (1789)	8324 (2109)
Energy (kcal)	I	2105 (583)	1916 (782)	1823 (608)
	C	2058 (644)	1976 (428)	1992 (505)
Protein (g)	I	80.2 (26.4)	73.6 (26.8)	68.3 (22.4)
	C	77.4 (27.1)	72.3 (12.4)	74.7 (20.3)
Carbohydrate (g)	I	235.1 (63.4)	241.0 (103.4)	209.3 (68.0)
	C	238.3 (74.5)	232.4 (65.7)	222.4 (56.5)
Fat (g)	I	89.8 (31.4)	71.9 (37.6)	76.0 (35.8)
	C	78.4 (33.3)	77.3 (22.8)	78.4 (23.9)
MUFA (g)	I	29.6 (13.3)	24.1 (15.4)	26.1 (14.2)
	C	24.9 (12.9)	24.3 (7.5)	25.5 (7.8)
PUFA (g)	I	13.6 (5.5)	10.2 (4.9)*	9.6 (3.9)
	C	11.6 (6.9)	12.4 (6.7)	11.2 (6.4)
SATFA (g)	I	32.1 (13.6)	27.1 (17.2)	28.8 (17.6)
	C	28.0 (13.5)	27.7 (9.2)	29.1 (9.5)
Cholesterol (mg)	I	272.2 (146.3)	206.2 (101.8)	218.9 (103.6)*
	C	227.7 (88.4)	241.3 (96.8)	262.3 (82.3)
Total sugar	I	89.8 (29.7)	127.5 (45.2) ***	91.1 (45.9)
	C	105.3 (44.8)	97.1 (43.8)	94.1 (30.3)
NMES	I	61.8 (27.2)	81.2 (39.1)*	62.8 (38.1)
	C	72.3 (31.4)	67.0 (31.4)	61.5 (22.9)
Starch	I	136.99 ± 48.28	106.61 ± 61.27*	107.32 ± 33.92
	C	127.00 ± 49.46	124.94 ± 42.90	114.95 ± 37.04
Dietary fibre	I	18.28 ± 5.62	21.18 ± 8.32	16.17 ± 4.61
	C	17.80 ± 5.79	18.03 ± 4.92	17.59 ± 5.25
NSP	I	12.69 ± 4.10	16.91 ± 6.30***	12.58 ± 3.51
	C	13.53 ± 5.16	12.69 ± 3.78	12.93 ± 4.42
Retinol (µg)	I	303.2 (149.6)	264.8 (214.1)*	321.9 (286.9)
	C	263.4 (119.5)	344.9 (228.5)	392.6 (210.1)
β-carotene (µg)	I	1576.1 (902.7)	6056.1 (2113.8)***	2057.0 (1478.6)
	C	2201.8 (1846.8)	1942.5 (974.5)	1504.4 (1206.2)
Vitamin B12 (µg)	I	4.41 (2.76)	4.00 (2.90)	3.29 (1.89)
	C	4.53 (3.13)	4.03 (1.96)	4.27 (1.98)
Folate (µg)	I	238.4 (91.6)	352.3 (146.6)**	233.68 ± 106.21
	C	264.8 (83.0)	274.5 (86.4)	243.2 (79.4)
Vitamin C (mg)	I	70.70 (36.72)	247.68 (78.05)***	103.92 (67.00)
	C	77.38 (44.66)	82.16 (49.00)	89.15 (49.78)
α-tocopherol (mg)	I	7.38 (3.63)	7.77 (3.40)	7.17 (3.15)
	C	6.69 (3.28)	7.14 (3.31)	5.86 (3.59)
Iron (mg)	I	14.06 (8.92)	14.54 (9.23)	12.47 (6.55)
	C	13.2 (5.9)	12.5 (3.2)	13.7 (7.5)
Potassium (mg)	I	2928 (722)	3518 (1152)**	2598 (731)
	C	3118 (837)	2957 (697)	2899 (759)
Manganese (mg)	I	3.5 (1.6)	3.4 (1.6)	3.4 (1.5)
	C	3.4 (1.4)	3.1 (1.1)	3.0 (1.2)
Magnesium (mg)	I	274.2 (93.9)	267.8 (97.1)	249.5 (89.2)
	C	293.7 (96.6)	280.4 (53.9)	265.9 (61.6)
Phosphorus (mg)	I	1298 (415)	1189 (445)	1111 (359)
	C	1294 (381)	1243 (271)	1253 (340)
Calcium (mg)	I	821.9 (267.1)	744 (302)	692 (317)
	C	876.8 (328.8)	843 (300)	816 (323)
Zinc (mg)	I	9.0 (4.4)	7.3 (3.2)	7.5 (3.0)
	C	8.9 (4.6)	7.9 (1.8)	8.1 (2.1)

*C* control (*n* = 21–24), *I* intervention (*n* = 19–21). Monounsaturated fatty acids (MUFA); polyunsaturated (PUFA) fatty acids; saturated fatty acids (SATFAs); non-milk extrinsic sugars (NMES); non-starch polysaccharides (NSP); intake was measured before (pre-run-in), at the end of the intervention (week 12), and post-washout. Results are mean dietary intake (SEM) calculated from analysis of 4-day weighed food diaries

* *P* < 0.05, ** *P* < 0.01, *** *P* < 0.001 refer to significant differences between treatment groups

**Table 3:** Plasma concentrations of vitamins B2, B9 (folate), B12 and C, retinol, carotenoids and tocopherols by intervention group

Biomarker	Group	Pre-run-in	Baseline	Week 4	Week 8	Week 12	Post-washout
Plasma folate	C	6.6 (0.5)	6.4 (0.5)	6.9 (0.5)	6.8 (0.6)	7.1 (0.6)	6.8 (0.6)
	I	6.4 (0.3)	6.1 (0.3)	6.9 (0.4)	7.1 (0.4)	7.5 (0.3)**	7.1 (0.5)*
Red cell folate	C	1038 (82)	10,267 (85)	1091 (101)	1065 (95)	1068 (95)	1135 (103)
	I	1108 (73)	1121 (79)	1212 (76)	1213 (75)**	1279 (85)**	1322 (80)*
Vitamin B2	C	14.0 (4.1)	14.7 (3.8)	16.1 (3.9)	17.1 (4.6)	17.9 (4.9)	16.3 (3.9)
	I	10.8 (1.5)	8.8 (1.6)	9.9 (1.7)	9.8 (1.6)	10.0 (1.6)	9.6 (1.5)
Vitamin B12	C	340 (25)	315 (23)	322 (24)	311 (26)	323 (25)	323 (27)
	I	318 (26)	322 (30)	310 (30)	295 (28)	297 (31)	293 (27)
Vitamin C	C	46.5 (4.6)	50.1 (4.9)	56.1 (4.1)	56.6 (3.5)	59.9 (4.1)	53.2 (3.4)
	I	55.1 (4.9)*	49.1 (4.1)	72.1 (4.6)**	70.3 (5.1)**	68.7 (4.9)*	60.7 (4.9)*
Retinol	C	0.52 (0.02)	0.48 (0.02)	0.49 (0.02)	0.50 (0.02)	0.49 (0.02)	0.48 (0.02)
	I	0.49 (0.04)	0.52 (0.04)	0.53 (0.03)	0.53 (0.03)	0.53 (0.03)	0.52 (0.03)
α-tocopherol	C	11.8 (0.4)	11.1 (0.3)	11.6 (0.6)	11.8 (0.5)	11.2 (0.5)	11.2 (0.4)
	I	11.8 (0.6)	11.4 (0.5)	11.5 (0.6)	11.4 (0.5)	11.7 (0.6)	11.6 (0.6)
γ-tocopherol	C	0.75 (0.06)	0.66 (0.04)	0.68 (0.06)	0.73 (0.09)	0.71 (0.07)	0.73 (0.05)
	I	0.59 (0.04)*	0.65 (0.16)	0.59 (0.05)	0.60 (0.07)	0.63 (0.06)	0.67 (0.05)
β-carotene	C	0.28 (0.04)	0.28 (0.04)	0.29 (0.04)	0.29 (0.04)	0.30 (0.04)	0.29 (0.04)
	I	0.26 (0.04)	0.23 (0.04)	0.42 (0.06)***	0.42 (0.06)***	0.42 (0.07)**	0.30 (0.06)
α-carotene	C	0.07 (0.01)	0.06 (0.01)	0.07 (0.01)	0.06 (0.01)	0.06 (0.01)	0.07 (0.01)
	I	0.07 (0.01)	0.06 (0.01)	0.09 (0.01)***	0.10 (0.01)***	0.11 (0.02)**	0.08 (0.02)
β-cryptoxanthin	C	0.12 (0.03)	0.11 (0.02)	0.11 (0.03)	0.13 (0.04)	0.12 (0.03)	0.11 (0.02)
	I	0.12 (0.02)	0.11 (0.02)	0.13 (0.01)*	0.13 (0.01)	0.13 (0.01)	0.10 (0.01)
Lycopene	C	0.48 (0.05)	0.46 (0.04)	0.47 (0.05)	0.52 (0.05)	0.50 (0.04)	0.50 (0.04)
	I	0.45 (0.05)	0.54 (0.08)	0.44 (0.04)	0.42 (0.03)*	0.44 (0.05)**	0.53 (0.05)
Lutein/zeaxanthin	C	0.15 (0.02)	0.15 (0.01)	0.15 (0.01)	0.15 (0.01)	0.16 (0.01)	0.16 (0.02)
	I	0.14 (0.01)	0.15 (0.01)	0.26 (0.02)***	0.25 (0.02)***	0.25 (0.02)***	0.17 (0.02)

Plasma folate; ng/ml, red cell folate; ng/mg Hb, B2; pmol/ml, B12; pg/ml, vitamin C, μM; α-carotene, β-carotene, lutein/zeaxanthin, lycopene, retinol, α- and γ-tocopherol; μg/ml

Results are mean ± (SEM)

C control (*n* = 21–24), *I* intervention (*n* = 19–21)

* *P* < 0.05, ** *P* < 0.01, *** *P* < 0.001 refer to significant differences between treatment groups

**Table 4:** Antioxidant capacity in whole-blood, plasma and lymphocytes by intervention group

Biomarker	Group	Pre-run-in	Baseline	Week 4	Week 8	Week 12	Post-washout
TEAC	C	1240 (43)	1199 (60)	1274 (54)	1071 (89)	1201 (70)	1262 (61)
	I	1294 (70)	1326 (64)	1392 (58)	1367 (50)*	1387 (52)	1284 (56)
HORAC	C	38,612 (1397)	39,319 (1528)	38,239 (1599)	37,145 (1540)	35,597 (1434)	35,961 (1683)
	I	33,023 (2135)*	35,160 (2595)	33,068 (2000)	33,359 (2171)	31,483 (2173)	30,551 (2057)
FRAP	C	1329 (102)	1330 (104)	1290 (95)	1388 (102)	1362 (103)	1360 (104)
	I	1395 (103)	1393 (100)	1409 (93)	1471 (97)	1453 (112)	1422 (98)
Endogenous DNA breakage	C	90.9 (7.5)	112.8 (8.0)	132.4 (10.9)	117.9 (11.9)	109.3 (13.6)	103.2 (7.9)
	I	98.2 (6.2)	139.6 (15.8)	135.0 (10.8)	114.9 (9.7)	102.7 (12.4)	109.6 (7.5)
H_2_O_2_-induced DNA breaks	C	105.7 (6.2)	105.6 (8.1)	101.1 (7.6)	98.8 (5.4)	101.3 (5.6)	107.3 (6.9)
	I	98.3 (6.6)	99.1 (8.5)	89.5 (6.4)	93.5 (7.1)	98.3 (7.4)	111.5 (5.8)
Catalase	C	2295 (98)	2323 (137)	2357 (121)	2403 (126)	2311 (107)	2220 (118)
	I	2276 (109)	2254 (110)	2188 (121)	2235 (117)	2263 (134)	2200 (110)
GSHPx	C	32.3 (1.5)	31.4 (1.2)	32.1 (1.5)	31.9 (1.3)	31.1 (1.3)	29.0 (1.2)
	I	32.7 (1.4)	31.8 (1.4)	31.8 (1.4)	32.2 (1.5)	31.4 (1.4)	30.9 (1.4)
SOD	C	1814 (62)	1843 (71)	1923 (72)	1924 (67)	1933 (81)	1930 (82)
	I	1706 (52)	1752 (55)	1784 (60)	1779 (48)	1828 (55)	1806 (57)

TEAC; μmol Trolox equivalents/L, FRAP; μmol Fe(II)/L, HORAC; net AUC, catalase, GSHPx, SOD; (U/g Hb), endogenous and oxidative DNA damage; arbitrary units

Results are mean ± (SEM)

*C* control (*n* = 21–24), *I* intervention (*n* = 19–21)

* *P* < 0.05 refers to significant differences between treatment groups

**Table 5:** Plasma concentrations of glucose, homocysteine, lipids and pulse, blood pressure (BP) and arterial stiffness, by intervention group

Biomarker	Group	Pre-run-in	Baseline	Week 4	Week 8	Week 12	Post-washout
Glucose (mmol/L)	C	5.33 (0.09)	5.25 (0.11)	5.18 (0.09)	5.21 (0.10)	5.15 (0.07)	5.21 (0.10)
	I	5.33 (0.12)	5.41 (0.09)	5.39 (0.08)	5.37 (0.09)	5.42 (0.10)	5.37 (0.10)
Homocysteine (μM)	C	9.53 (0.46)	9.51 (0.64)	9.18 (0.55)	9.34 (0.63)	9.67 (0.60)	9.31 (0.58)
	I	10.19 (0.45)	10.89 (0.55)	10.36 (0.48)	10.64 (0.55)	10.43 (0.66)	10.84 (0.63)
Cholesterol (mmol/L)	C	5.39 (0.18)	5.06 (0.13)	5.15 (0.17)	5.23 (0.17)	5.12 (0.18)	5.29 (0.18)
	I	5.19 (0.22)	5.21 (0.20)	5.24 (0.26)	5.26 (0.23)	5.37 (0.24)	5.41 (0.25)
HDL (mmol/L)	C	1.36 (0.08)	1.33 (0.07)	1.37 (0.09)	1.37 (0.09)	1.38 (0.08)	1.37 (0.10)
	I	1.36 (0.08)	1.39 (0.09)	1.32 (0.09)	1.37 (0.09)	1.32 (0.08)	1.39 (0.08)
LDL (mmol/L)	C	3.23 (0.17)	2.93 (0.14)	2.97 (0.14)	3.02 (0.17)	2.99 (0.16)	3.10 (0.15)
	I	2.96 (0.19)	3.04 (0.20)	3.08 (0.23)	3.09 (0.22)	3.22 (0.24)	3.19 (0.23)
TG (mmol/L)	C	1.22 (0.16)	1.30 (0.16)	1.26 (0.18)	1.31 (0.17)	1.09 (0.09)	1.29 (0.16)
	I	1.29 (0.19)	1.19 (0.15)	1.28 (0.22)	1.27 (0.16)	1.31 (0.18)	1.23 (0.19)
NEFA (mmol/L)	C	0.51 (0.03)	0.55 (0.06)	0.54 (0.04)	0.56 (0.06)	0.51 (0.03)	0.48 (0.03)
	I	0.52 (0.03)	0.50 (0.05)	0.48 (0.04)	0.54 (0.05)	0.49 (0.04)	0.51 (0.04)
BP (systolic; mmHg)	C	122.8 (2.5)	123.6 (2.5)	123.8 (2.7)	120.4 (2.4)	122.5 (2.2)	120.2 (2.2)
	I	124.9 (2.3)	124.1 (2.2)	126.7 (2.3)	123.7 (2.0)	120.8 (1.5)	123.4 (2.0)
BP (diastolic)	C	78.9 (1.8)	78.7 (1.6)	75.6 (1.7)	78.8 (1.7)	79.3 (1.5)	76.1 (1.5)
	I	81.4 (1.4)	80.0 (1.3)	78.8 (1.4)	80.4 (1.4)	79.2 (1.1)	80.4 (1.5)
Heart rate (BPM)	C	65.2 (1.7)	62.5 (2.4)	62.2 (1.9)	63.4 (1.9)	64.1 (1.8)	61.91 (2.1)
	I	65.7 (2.2)	66.2 (2.3)	65.6 (1.9)	69.3 (2.1)	67.2 (1.8)	66.7 (2.0)
PWV (HR; M/s)	C	65.7 (1.9)	ND	ND	ND	65.5 (1.9)	ND
	I	68.0 (2.0)	ND	ND	ND	68.6 (1.9)	ND
PWV (SI; M/s)	C	9.5 (0.5)	ND	ND	ND	9.1 (0.3)	ND
	I	8.6 (0.4)	ND	ND	ND	8.8 (0.5)	ND
PWV (RI; M/s)	C	72.8 (2.1)	ND	ND	ND	69.7 (2.3)	ND
	I	64.6 (3.0)	ND	ND	ND	62.6 (3.7)	ND
PWV (PPT; M/s)	C	191.7 (8.9)	ND	ND	ND	203.5 (9.9)	ND
	I	204.5 (7.9)	ND	ND	ND	203.8 (9.1)	ND

Results are mean ± (SEM)

*C* control (*n* = 21–24), *I* intervention (*n* = 19–21), *BP* blood pressure, *PWV* pulse wave velocity, *HR* heart rate, *SI* stiffness index, *RI* reflection index, *PPT* point-to-point time, *ND* not determined

### Effect of intervention on fruit and vegetable intake (compliance)

**Fig. 2:**
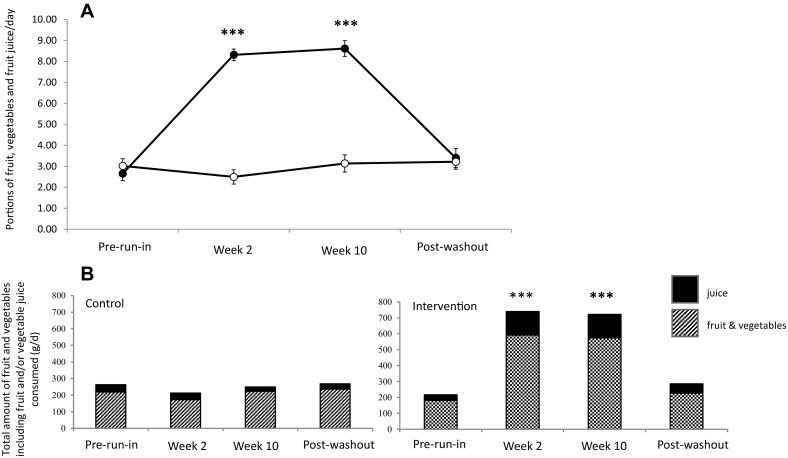
Effect of intervention on number of portions (**a**) and fruit, and vegetable and juice intake (**b**). **a** Values are mean portions consumed daily ± SEM; control (*open circles*, *n* = 24) and intervention (*closed circles*, *n* = 21). **b** Relative fruit and vegetable, and juice intake, in each treatment group. Intake was measured before (pre-run-in), throughout the intervention (week 2 and week 12), and post-washout. ***P < 0.0001 refers to significant differences between groups

Fruit, fruit juice and vegetable consumption, estimated using 4-day weighed intake data, were similar between the treatment groups prior to the start of the study (mean 3.02 and 2.63 portions per day for the control and intervention group, respectively; *P* > 0.05) and remained essentially unchanged in the control group throughout the intervention (Fig. 2). Participants in the intervention group were asked to eat all of the additional juice, fruits and vegetables provided to them, ensuring equal consumption of fruits and vegetables across the study. Intake of fruit, juice and vegetables increased significantly (*P* < 0.001) by approximately five portions in the intervention group to a maximum of 8.4 portions per day after 12 weeks (Fig. 2). Post-washout, the intake of fruit, vegetables and juices in the intervention group returned to almost pre-intervention levels (3.33 portions) and was not significantly different from baseline intake (*P* > 0.05).

### Effect of intervention on macro- and micronutrient intake

Intervention had no significant effect on participant weight, total energy, total protein, total carbohydrate or total fibre intake (Table 2). While consuming up to eight portions of fruits and vegetables daily did not influence total fat intake, PUFA intake was significantly lower in the intervention group at the end of the 12-week intervention (approx. 25%), but returned to pre-study levels during the washout (Table 2). Similarly, total cholesterol intake was lowered by eating additional fruits, vegetables and juices (Table 2), and this effect was sustained post-washout (approx. 20%). Total sugar and NMES intake increased in the intervention group after 12 weeks on the study (approx. 40 and 30%, respectively). Total starch intake decreased (approx. 20%) and NSP intake increased (approx. 25%) in the intervention group (Table 2). None of these changes were maintained after the 6-week washout (Table 2). Intake of the antioxidants vitamin C and β-carotene increased significantly in participants eating additional fruits and vegetables after 12 weeks on study (3.5- and 3.8-fold, respectively), as did the intake of folate (approx. 50%). These changes were not maintained post-intervention (Table 2). Retinol intake was slightly lower in the intervention group 12 weeks into the study (approx. 13%), but returned to pre-intervention levels during the washout phase (Table 2). Vitamin B12 and α-tocopherol intake were not altered by intervention (Table 2). Potassium intake was increased in response to increasing fruit, vegetable and juice consumption (approx. 20%), but this was not maintained (Table 2). Intake of iron, manganese, magnesium, phosphorous, calcium and zinc were unaffected by intervention (Table 2).

### Blood nutrient status

**Fig. 3:**
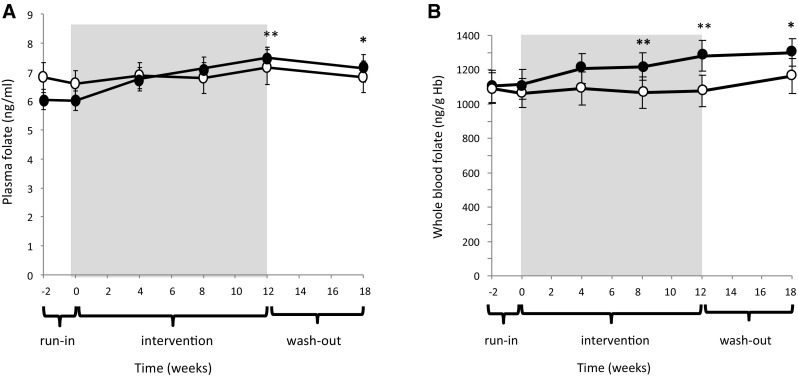
Plasma (**a**) and whole-blood (**b**) folate by intervention group. Results are mean ± SEM. Control (*open circles,* *n* = 21–24) and intervention (*closed circles*, *n* = 19–21). *P < 0.05, **P < 0.01 refer to significant differences between groups

**Fig. 4:**
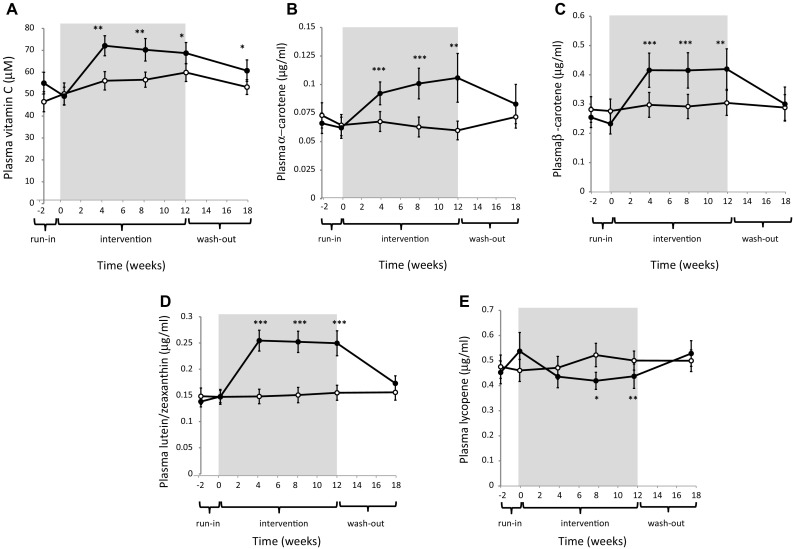
Plasma vitamin C (**a**), a-carotene (**b**), b-carotene (**c**), lutein/zeaxanthin (**d**) and lycopene (**e**) by intervention group. Results are mean ± SEM. Control (*open circles*, *n* = 21–24) and intervention (*closed circles*, *n* = 19–21). *P < 0.05, **P < 0.01, ***P < 0.001 refer to significant differences between groups

Providing volunteers an additional 480 g of fruit and vegetables and 300 ml of fruit juice for 12 weeks significantly (and in some cases progressively) increased the circulating concentrations of folate (approx. 15% plasma and whole blood; Fig. 3), vitamin C (approx. 35%), and the carotenoids, α-carotene (approx. 50%), β-carotene (approx. 70%) and lutein/zeaxanthin (approx. 70%, Fig. 4). No differences in plasma retinol, α- and γ-tocopherol and plasma B2 or B12 were observed (Table 3). Plasma lycopene declined significantly (approx. 10%) in the treatment group after 4 weeks on the intervention and remained lower at week 12. At the end of the washout period, plasma α-carotene, β-carotene, lycopene and lutein/zeaxanthin concentrations had returned to baseline, with no significant differences measured between the treatment groups. Conversely, plasma and whole-blood folate and plasma vitamin C levels remained slightly but significantly elevated in the intervention group (Figs. 3, 4).

### Antioxidant capacity, antioxidant enzyme activity and oxidative DNA damage

The effect of intervention on several plasma and intracellular markers of antioxidant capacity and resistance to oxidative stress was measured. Substantially increasing intake of fruits, fruit juices and vegetables (up to a total of 8.4 portions) in general had no significant effect on plasma antioxidant capacity (TEAC, HORAC and FRAP), nor on the ability of isolated lymphocytes to resist DNA damage in response to an oxidative stress ex vivo (Table 4). Moreover, the intervention had no significant effect on the endogenous enzymatic antioxidant defence system, with no significant changes in the activity of erythrocyte catalase, glutathione peroxidase and superoxide dismutase (Table 4).

### Plasma glucose, homocysteine, lipids, blood pressure and arterial stiffness

Despite significantly increasing circulating folate levels, increasing fruit, fruit juice and vegetable intake substantially did not change plasma total homocysteine concentrations (Table 4). Nor did it alter plasma glucose or lipid status [total cholesterol, HDL, LDL, TG, non-esterified fatty acids (NEFA)] or several markers of vascular health including BP, heart rate and vascular tone (Table 5). Moreover, there were no significant changes in body weight in response to intervention (Table 5). However, it should be noted that these markers of vascular health were not the primary end points of this study and accordingly the study was not powered specifically to detect changes in these biomarkers.

### Attitudes towards increasing and sustaining fruit and vegetable intake

Attitude of the participants to eating fruits and vegetables and their perceived benefits and barriers to sustaining a high intake of fruits and vegetables were assessed using a questionnaire. Thirty-two of the 45 original participants completed the questionnaires (71% response rate), with an equal number of responders in both study groups (*n* = 16).

All of the respondents were aware of the UK Government dietary recommendation of “5-a-day” guidelines for fruit and vegetable consumption. The majority reported their consumption of fruit and vegetables 12 months after the study as 3–4 portions per day or less. No difference in intake was found between the intervention and control group. 62.5% of those in the intervention group believed that they consumed more fruits and vegetables 12 months post-intervention compared with before the study, while 37.5% said they ate approximately the same amount. The majority of the control group (62.5%) felt they consumed about the same. Of those that said their intake had increased, the main reasons given included (1) they enjoyed eating fruit and vegetables; (2) they felt better after eating fruits and vegetables, or (3) they wanted to eat a better diet. Only one respondent reported that their intake had decreased, and this was reported to be due to cost.

When comparing the amount of fruit and vegetables they ate 12 months post-intervention with during the study, 68.8% of people in the intervention group reported that they consumed less, while 62.5% of the control group reported that they consumed about the same. The main reasons for decreasing their intake included (1) inconvenience (e.g. during the study all fruit, juices and vegetables were delivered to them); (2) there was too much fruit and vegetables to eat during the study; (3) variation in amount eaten seasonally (i.e. they tended to eat more in the summer) and (4) cost.

The majority of respondents in both treatment groups agreed that eating more fruit and vegetables meant that they ate fewer other foods, that fruit and vegetables were filling, that they liked most fruit and vegetables and they did not think that fruit and vegetables were boring. Twelve months after the intervention, fewer participants in the intervention group agreed with the statement ‘I think that eating 5-a-day is easy’ compared with the control group (Table 6).

**Table 6:** Attitudes to fruit and vegetable consumption

Thinking about eating fruit and vegetables, how much do you agree or disagree with these statements?	Group	Agree	Neither agree nor disagree	Disagree
I think that eating 5-a-day is easy	C	43.8	25.0	31.3
	I	26.7	40.0	33.3
Eating more fruit and vegetables means I eat fewer other foods	C	68.8	18.8	12.5
	I	81.3	0	18.8
I find fruit and vegetables filling	C	62.6	25.0	12.5
	I	56.3	31.1	12.5
I find fruit and vegetables boring	C	6.3	12.5	81.3
	I	6.3	25.0	68.8
I like most fruit and vegetables	C	93.8	0	6.3
	I	75.1	0	18.8

Results are expressed as percentage (%) of participants (C *n* = 16; I *n* = 16)

The majority of both the intervention and control group believed that they should eat more fruit and vegetables (87.5 and 75.0%, respectively).

With regard to perceived barriers to eating fruit and vegetables regularly, 56.3% of the intervention group and 46.7% of the control group believed that there were obstacles to incorporating more fruit and vegetables into the diet. The most common reasons were (1) the cost of fruit (but not vegetables); (2) the short shelf life of fruit and vegetables and (3) having to shop more frequently (Table 7). A lack of knowledge of how to prepare vegetables or time to prepare them was not reported as a barrier. Moreover, convenience, availability, lack of cooking skills, preparation time and not being accustomed to eating fruit and vegetables were not perceived as major obstacles to consuming fruits and vegetables by either group.

**Table 7:** Barriers to fruit and vegetable consumption

Thinking about things that stop you eating more fruit and vegetables, how much do you agree or disagree with these statements?	Group	Agree	Neither agree nor disagree	Disagree
I think buying fruit is expensive	C	62.5	31.3	6.3
	I	62.5	12.5	25.0
I think buying vegetables is expensive	C	43.8	43.8	12.5
	I	18.8	31.3	50.0
I have to go to the shops more often to buy fruit and vegetables	C	40.0	33.3	26.7
	I	68.8	12.5	18.8
Fruit is not convenient to eat	C	0	12.5	87.5
	I	0	12.5	87.5
There is nowhere handy for me to buy fruit and vegetables	C	0	12.5	87.5
	I	12.6	6.3	76.8
I don’t know how to cook vegetables	C	6.3	0	93.8
	I	6.3	12.5	81.3
I think that preparing and cooking vegetables to eat takes too much time	C	12.5	6.3	81.3
	I	13.3	13.3	73.3
I am not in the habit of eating fruit and vegetables	C	6.3	18.8	75.1
	I	12.5	18.8	68.8
Fruit and vegetables do not keep fresh/stored for very long	C	56.2	18.8	25.0
	I	43.8	31.3	25.0

Results are expressed as percentage (%) of participants (C *n* = 16; I *n* = 16)

Taste was the most commonly reported influence on purchasing fruit and vegetables in both groups. The least common influences were the provenance of the fruits and vegetables and whether it was organic or not (Table 8).

**Table 8:** Choosing fruits and vegetables

When choosing fruit and vegetables to buy, how much do the following influence your choice?	Group	A lot	Some	A little/not at all
Price of fruit and vegetables	C	50.0	25.0	25.0
	I	68.8	18.8	12.5
Taste of fruit and vegetables	C	81.2	18.8	0
	I	87.5	6.3	6.3
Appearance of the fruit or vegetables	C	62.5	18.8	18.8
	I	50.0	31.3	18.8
If the fruit or vegetable is on special offer	C	56.2	31.3	12.5
	I	75.0	25.0	0
If the fruit or vegetable is in season	C	50.0	37.5	12.5
	I	71.4	28.6	0
If the fruit or vegetable is grown in Scotland	C	18.8	31.3	50.0
	I	18.8	31.3	50.0
If the fruit or vegetable is organic	C	12.5	6.3	81.2
	I	18.8	12.5	68.8
What your family prefer to eat	C	56.2	12.5	31.3
	I	37.5	18.8	43.8

Results are expressed as percentage (%) of participants (C *n* = 16; I *n* = 16)

## Discussion

Numerous human observational studies report that high intakes of fruits and vegetables are linked with a lower risk of chronic human diseases including cancer, vascular disease and diabetes [1–5]. While, many global organisations (WHO) and national government initiatives encourage consumers to consume a minimum of five portions or 400 g of fruit and vegetables per day [6, 18], recommendations for intake vary globally. A recent meta-analyses of 95 prospective studies reported a significantly reduced relative risk for CVD, stroke, total cancer incidence and all-cause mortality with fruit and vegetables intakes in excess of 200 g daily [5]. Critically, this meta-analysis, which analysed data from a much larger number of studies than previous investigations, reported a dose–response relationship for intake, disease and death, with consumption of 800 g of fruit and vegetables per day (10 portions) considered optimal [5].

Here, the average reported intake of fruits, juices and vegetables at the start of the intervention was approx. 240 g each day, substantially below the recommended daily intake. Data from national surveys worldwide indicate that a large proportion of the general populace does not meet the guideline intake for fruits and vegetables [6, 18]. In a postal survey of 1069 men and women (aged 20–88 years) in the USA, 45% reported eating no fruit and 22% ate no vegetables daily [46]. More recent data from the NHANES survey (1988–2002) indicate that only 11% of American adults meet the current recommendations [47]. Intake data gathered from several European national food surveys and compiled by the European Food Safety Authority (EFSA), show that fruit and vegetable consumption averages 386 g per person per day in Europe [48]. However, of the 16 EU countries sampled, only 4 achieved the recommended daily intake of greater than 400 g of fruit and vegetables, with 75% of countries failing to meet the WHO and national guidelines. In good agreement with the findings from the current study, average consumption in the UK was 258 g per day [48]. Whilst there has been a slight increase in reported fruit and vegetable consumption in the period since 2001, only a minority (approx. 30%) of the UK population are meeting dietary guidelines [9, 49]. Failure to eat sufficient fruits and vegetables in order to maintain good health and prevent disease has been estimated to contribute 2.4% directly to the estimated overall burden of disease across Europe [50].

Compliance in this study, measured by dietary reporting (check list and 4-day weighed intake dairies) and plasma nutrient levels, was good, with the two treatment groups well differentiated in terms of fruit and vegetable consumption. The average intake was increased by 5 portions to a maximum of eight servings each day in the treatment group 12 weeks into the intervention. Consuming additional fruit, vegetables and fruit juice had no effect on calculated total energy, total fat, carbohydrate, protein or fibre intake, and a limited and transient effect on specific carbohydrate and lipid intake [starch, non-starch polysaccharides (NSP), total cholesterol and polyunsaturated fatty acid (PUFA) intakes], indicating no substantial displacement of habitual foods from the diet of the participants. As expected, antioxidant nutrient (vitamin C, β-carotene), folate and potassium intake increased substantially following consumption of up to 8.4 portions of fruits, vegetables and fruit juices daily for 12 weeks, suggesting potential beneficial effects of the intervention. The changes in nutritional micro- and macro-nutrient intakes described here agree well with findings from similar intervention studies [19, 44, 51–53]. Vitamin C, total carotene, β-carotene, potassium and NSP intake were significantly increased in volunteers fed five portions of fruit and vegetables for 8 weeks [51, 53]. Feeding volunteers an additional six portions of fruits and vegetables for 6 weeks significantly increased vitamin C, total carotenes, folate and NSP intake, measured by 24-h dietary recall [52]. Vitamin C intake was also increased in subjects asked to adhere to a Mediterranean style diet for 2 months [54].

Less positively, NMES and total sugar intake also increased significantly in this study, probably reflecting the increased intake of fruit and fruit juice in the treatment group. The potential deleterious impact of increased NMES, sugars and calories from consuming fruit juice (particularly 100% fruit juice) to excess, is of public interest, particularly with regard to childhood obesity [55, 56]. Similar changes in total sugar, NMES and dietary fructose, glucose, maltose and galactose intake in response to substantially increasing fruit, juice and vegetable consumption have been reported previously [51–53].

There was, in general, good agreement observed between calculated dietary intake and measured plasma nutritional biomarkers. Consuming approx. 8 portions of fruits, vegetables and fruit juices daily for 12 weeks significantly increased blood folate (plasma and whole blood), vitamin C, the carotenoids α- and β-carotene and lutein/zeaxanthin in healthy men and women who habitually consumed a diet low in these foods prior to intervention. Post-washout changes in circulating nutrient status are consistent with those described in other studies. Feeding subjects considered to be at risk of developing CVD up to 6 additional portions of fruits and vegetables for 18 weeks increased plasma folate, vitamin C, total flavonoid and carotenoid levels [52]. As here, changes in circulating nutrients broadly reflected increased estimated intake [52]. Similarly, plasma from participants fed the equivalent of 5 portions of fruits and vegetables per day in the form of soups, juices and “shots” prepared from carrot, tomato, red peppers, apple, strawberries, orange, banana and cherries for 4 weeks had elevated α-carotene and β-carotene levels. Total carotenoid and vitamin C intake [53] were also increased. In contrast to the findings from our study, plasma lycopene increased (31%). This probably reflects differences in foodstuffs consumed during the intervention and suggests that tomatoes may have been displaced from the participants’ diet in this study. In a similar study, plasma β-carotene was elevated in volunteers fed 600 g/day of fruits vegetables and orange juice for 4 weeks [57]. However, plasma vitamin C was unchanged, possibly due to a shorter intervention and a more restricted variety of foods [57]. Increasing blood and dietary folate intake is associated with a decreased risk of cancer, CVD and stroke [28, 58]. In this study, participants consuming up to 8 portions of fruits, juices and vegetables daily for 12 weeks reported significantly increased plasma and whole-blood folate (a marker of long-term folate intake). An improvement in folate status was similarly observed in healthy men and women consuming fruits and vegetables containing 350 μg of folate each day for 4 weeks [20]. Conversely, subjects eating 200 g of fruits and vegetables with a folate content of approx. 40 μg/day for an equivalent time period did not significantly increase blood folate concentrations [59].

Increasing fruit and vegetable consumption in this study was not found to affect measures of plasma antioxidant capacity or cellular antioxidant function. Fruits and vegetables contain high levels of antioxidant vitamins and phytochemicals including phytophenols that enhance antioxidant status and oxidative stress resistance in vitro and in vivo [25–27]. Evidence, primarily from in silico and in vitro studies, has demonstrated these compounds to be powerful scavengers of ROS. Oxidative stress, which occurs when the formation of ROS and other radical species overwhelms the circulating and intracellular defence systems, is implicated in the development of several human chronic diseases including diabetes, CVD, stroke and cancer [1–4]. Here, antioxidant capacity (plasma HORAC, TEAC, and FRAP), antioxidant enzyme activity (red cell catalase, glutathione peroxidase and superoxide dismutase) were unaffected by intervention. Similarly, increasing fruit and vegetable consumption in excess of WHO and UK government recommendations, did not improve the resistance of lymphocytes isolated from the participants to an induced oxidative stress. The 4-day weighed intake data collected throughout this study, which showed a significant increase in reported fruit, juices and vegetable consumption of approximately 5 portions per day in the intervention group, together with the observed increases in circulating vitamins and carotenoids, indicate that the lack of effect of supplementation reported here was not a consequence of poor compliance (Fig. 1). Rather, it suggests that increasing fruit and vegetable consumption significantly increases the circulating concentrations of beneficial nutrients in healthy subjects without inducing a corresponding change in antioxidant capacity or markers of oxidative stress. The effect of consuming plant-based foods and beverages on plasma antioxidant capacity is inconsistent, despite significant conservation of the methodologies employed [57, 60].

A recent meta-analysis of more than 100 interventions found a generally positive effect of fruits, vegetables, plant foods, red wine and tea on plasma non-enzymatic antioxidant capacity (NEAC; [60]). Similarly, FRAP (which was used in this study) was elevated slightly (approx. 10%) in subjects at risk of CVD who consumed up to six additional portions of fruits and vegetables per day for up to 18 weeks [52]. Eating a Mediterranean style diet for 8 weeks, while significantly increasing total estimated vitamin C intake (approx. 30%) in subjects with abdominal obesity, also caused a small increase in plasma ORAC (approx. 8%) and a trend towards increased FRAP activity [54]. Equally, a null or highly limited effect of fruit and vegetable intervention on surrogate markers of disease risk has also been reported. Increasing fruit and vegetable intake had no effect on plasma antioxidant capacity (estimated by TEAC, FRAP, ORAC), erythrocyte SOD, catalase, glutathione reductase and GST activities (although GPX was significantly elevated), after consuming 600 g of fruit and vegetables per day for 24 days in a placebo-controlled intervention [57]. Leucocyte DNA single-strand breakage, DNA base oxidation damage (pyrimidines and purines), sensitivity to DNA oxidation damage and DNA repair enzyme efficacy (hOGG1 and ERCCI) were also unchanged [57]. A lack of effect on antioxidant capacity and cellular antioxidant activity was reported in a similar intervention (600 g of fruit and vegetables daily for 25 days), although biomarkers of protein and lipid oxidation did respond positively, indicating that specific molecular targets may react differently to intervention [61]. Conversely, consuming a diet supplemented for 8 weeks with antioxidant-rich plant-based foods had a significant positive effect on DNA single-strand breakage (20% reduction) and base excision repair capacity (40% increase), in a group of healthy men. Here, oxidised DNA base damage and resistance to oxidative DNA damage was unchanged by the intervention, while, inexplicably, nucleotide excision repair activity was substantially decreased (39%; [62]).

Interestingly, consuming non-nutritional quantities of a single foodstuff, or fruits and vegetables rich in a particular nutrient has been shown to alter the status of several of the biomarkers employed here. Feeding healthy subjects a single meal (200 g) of cooked onions which are high in flavonoids, significantly reduced DNA oxidation damage in lymphocytes collected from the volunteers [22]. Similarly, endogenous DNA damage and base excision DNA repair capacity were improved in lymphocytes from volunteers fed a large bolus of kiwi fruit puree (equivalent to three fruits) [21]. It has been suggested that antioxidants delivered in fluid-based interventions using wine, fruit and vegetable juices and purees, are more bioavailable than in complex food matrixes. Drinking red wine (375 ml/day for 2 weeks) was found to increase total plasma phenolic concentration and reduce markers of oxidation (TBARS and conjugated dienes) in oxidised LDL from healthy subjects [63]. Similarly, circulating vitamin C, α- and β-carotene were significantly higher post-supplementation in healthy participants fed an equivalent amount (400 g/day) of fruits and vegetables in liquid form compared with nutrient levels measured here [53]. In agreement with the findings from this study, feeding a liquid diet was not associated with any improvement in biomarkers of DNA damage, inflammation or oxidative stress [53]. Likewise, feeding healthy subjects 750 ml of cranberry juice daily for 2 weeks significantly increased plasma vitamin C concentrations (approx. 30%), but had no protective effect on blood antioxidant capacity (FRAP, catalase, SOD and GSHPx) or genomic stability (DNA oxidation damage in lymphocytes and urine) [64].

This study has limitations. Fasted blood was collected and it is possible that any bioefficacy of the fruit and vegetables consumed is short-lived and undetectable using the biomarkers employed here. Indeed, we have shown previously that ingesting cranberry juice significantly increases plasma antioxidant capacity over a few hours, but that this effect is transient [65]. Moreover, certain of the assays employed here, while sensitive to changes in global antioxidant capacity are not relevant for measuring the impact of fruits and vegetables on systemic inflammation or vascular health (e.g. CRP, IL-6, sICAM, sVCAM and PAI-1). However, as described earlier, this study was not powered to identify changes in these biomarkers. A primary aim of this study was to investigate the impact of increasing fruit, juice and vegetable intake in an average normal population. Consequently, heathy subjects, with no pathologies and not taking prescribed medication were purposefully recruited. Antioxidant activity (NEAC) has been found to respond to intervention more strongly in subjects displaying chronic disease risk factors when compared with healthy subjects [58]. Whether this intervention would have had a greater impact in subjects with a higher BMI or hypercholesterolemia/hyperhomocysteinemia and considered at greater risk of chronic diseases, remains to be established.

Consumption of fruits, vegetables and juices returned to almost baseline levels in the treatment group after the intervention. The slight increase reported was equivalent to only half of one portion in this group (2.63 portions increasing to 3.33 portions). This increase in intake agrees well with published studies, where the average improvement gained by interventions designed to foster a sustained increase in fruit and vegetable consumption was approximately one half serving more per day [46]. However, this figure is substantially lower than that reported by Cox et al., who observed a sustained increase of 4.6 portions, 12 months after the end of an 8-week study [51]. Long-term positive changes in fruit and vegetable consumption were also reported following a randomised controlled trial very similar in design to that described here [66]. Here, participants were asked to consume less than 2 (control) or more than 5 (intervention) portions of fruits, vegetables and juices daily for 16 weeks. Participants in the intervention group increased their intake to an average of 6.0 portions of fruits, vegetables and juices each day at the end of the study [66]. At follow-up, while fruit and vegetable intake in this group had dropped substantially to 3.6 portions/day, former volunteers were still consuming a significantly higher intake of fruits and vegetables 18 months after the intervention when compared with control participants (2.6 portions/day; [66]).

Despite evidence that the vast majority of consumers fail to meet national guidelines for fruit and vegetable intake, most people, including those in this study, are aware of government recommendations, believe that it is easy to eat a balanced heathy diet and, that as individuals, they do eat healthily [49, 66–68]. Here, all of the participants were aware of the “5 A Day” guidelines, and believed that it was important to increase their fruit and vegetable intake, indicating that nutritional knowledge is not in itself a barrier to consumption. The majority of participants in both groups reported liking fruits and vegetables post-intervention, although this was less evident in those that had received fruits and vegetables within the intervention. Critically, these participants perceived that it was more difficult to eat the recommended five servings per day than those in the control group who had maintained a habitual low intake of approx. 3 servings. This may be a reflection of having experienced eating in excess of eight portions a day compared with those in the control group. This agrees with earlier findings where participants reported greater difficulty in eating two servings of vegetables with a meal after they had taken part in a fruit and vegetable intervention [51, 67].

Primary reasons given for the reduction in intake in the treatment group post-study were that the amount of fruit and vegetables in the intervention had been too much to continue eating, that it was inconvenient to shop for fresh fruits and vegetables as frequently as required, and that it was too expensive to eat more than five serving of fruits and vegetables daily. It is worth remembering that all fruits and vegetables were provided cost-free and that food delivery was tailored as far as possible to the participants work schedule and availability throughout this study. Moreover, it is noteworthy that, once withdrawn (washout period) fruit and vegetable consumption quickly declined towards habitual intake levels. These findings should be incorporated in the design of future population-focused interventions to promote healthy eating.

## Conclusion

These findings support claims that providing fruits, vegetables and fruit juices to people significantly increases intake and circulating levels of several nutrients positively associated with human health. Moreover, this improvement in nutrient status was maintained throughout the period of supplementation of fruit and vegetables. However, these data do not support the hypothesis that improving the status of specific dietary nutrients, including antioxidant vitamins and phytochemicals, has, within this time frame, a substantial beneficial effect on circulating or intracellular measures of global antioxidant efficacy or cytoprotection in healthy people. This study also identifies some of the perceived barriers associated with achieving the recommended national guidelines for fruit and vegetable intake.

## Electronic supplementary material

Below is the link to the electronic supplementary material.

Supplementary material 1 (PDF 23 kb)

## References

[1] Slavin JL, Lloyd B (2012). Health benefits of fruits and vegetables. Adv Nutr.

[2] Bellavia A, Larsson SC, Bottai M (2013). Fruit and vegetable consumption and all cause mortality: a dose-response analysis. Am J Clin Nutr.

[3] Wang X, Ouyang Y, Liu J (2014). Fruit and vegetable consumption and mortality from all causes, cardiovascular disease and cancer: systematic review and dose-response meta-analysis of prospective cohort studies. BMJ.

[4] Choi Y, Lee JE, Bae J-M (2015). Vegetable intake but not fruit intake is associated with a reduction in the risk of cancer incidence and mortality in middle-aged Korean men. J Nutr.

[5] Aune D, Giovannucci E et al., (2017) Fruit and vegetable intake and the risk of all cardiovascular disease, total cancer and all-cause mortality-a systematic review and dose-response meta-analysis of prospective studies. Int J Epidemiol:1–28. doi:10.1093/ije/dyw319 10.1093/ije/dyw319 PMC5837313 28338764

[6] Hall JN, Moore S, Harper SB (2009). Global variability in fruit and vegetable consumption. Am J Prev Med.

[7] Lock K, Pomerleau J, Causer L, Ezzati M, Lopez AD, Rogers A, Murray CJL (2004). Low fruit and vegetable consumption. Comparative quantification of health risks: global and regional burden of disease attributable to select major risk factors.

[8] Scarborough P, Nnoaham KE, Clarke D (2012). Modelling the impact of a healthy diet on cardiovascular disease and cancer mortality. J Epidemiol Commun Health.

[9] Department of Health, National Diet and Nutrition Survey. http://transparency.dh.gov.uk/2012/07/25/ndns-3-years-report/. Accessed 23 March 2017

[10] Steinmetz KA, Potter JD (1991). Vegetables, fruit and cancer II. Mechanisms. Cancer Causes Control.

[11] Riboli E, Horel T (2003). Epidemiological evidence of the protective effect of fruit and vegetables on cancer risk. Am J Clin Nutr.

[12] Hung HC, Joshipura KJ, Jiang R (2004). Fruit and vegetable intake and risk of major chronic disease. J Natl Cancer Inst.

[13] Smith-Warner SA, Spiegelman D, Yaunn SS (2001). Intake of fruits and vegetables and risk of breast cancer: a pooled analysis of cohort studies. JAMA.

[14] Dauchet L, Amouyel P, Hercberg S (2006). Fruit and vegetable consumption and risk of coronary heart disease: a meta-analysis of cohort studies. J Nutr.

[15] He FJ, Nowson CA, Lucas M (2007). Increased consumption of fruit and vegetables is related to a reduced risk of coronary heart disease: meta-analysis of cohort studies. J Hum Hypertens.

[16] Oude G, Gelejinse JM, Kronhout D (2010). Raw and processed fruit and vegetable consumption and 10-year coronary heart disease incidence in a population-based cohort study in the Netherlands. PLoS One.

[17] Mizrahi A, Knekt P, Montonen J (2009). Plant foods and the risk of cerebrovascular diseases: a potential protection of fruit consumption. Brit J Nutr.

[18] Woodside JV, Young IS, McKinley MC (2013). Fruit and vegetable intake and risk of cardiovascular disease. Proc Nutr Soc.

[19] Carter P, Gray LJ, Troughton J (2010). Fruit and vegetable intake an incidence of type-2 diabetes mellitus: systematic review and meta-analysis. BMJ.

[20] Brouwer IA, van Dusseldorp M, West CE (1999). Dietary folate from vegetables and citrus fruit decreases plasma homocysteine concentrations in humans in a dietary-controlled trial. J Nutr.

[21] Collins AR, Harrington V, Drew J (2003). Nutritional modulation of DNA repair in a human intervention study. Carcinogenesis.

[22] Boyle SP, Dobson VL, Duthie SJ (2000). Absorption and DNA protective effects of flavonoid glycosides from an onion meal. Eur J Nutr.

[23] Stea TH, Mansoor MA, Wandel M (2008). Changes is predictors and status of homocysteine in young male adults after a dietary intervention with vegetables, fruit and bread. Eur J Nutr.

[24] Root MM, McGinn MC, Nieman DC (2012). Combined fruit and vegetable intake is correlated with improved inflammatory and oxidant status from a cross-sectional study in a community setting. Nutrients.

[25] Arts ICW, Hollman PCH (2005). Polphenols and disease risk in epidemiological studies. Am J Clin Nutr.

[26] Duthie SJ (2007). Berry phytochemicals, genomic stability and cancer: evidence for chemoprotection at several stages in the carcinogenic process. Mol Nutr Food Res.

[27] Hollman PCH, Cassidy A, Comte B, Heinonen M, Richelle M, Richling E, Serafini M, Scalbert A, Sies H, Vidry S (2011) The biological relevance of direct antioxidant effects of polyphenols for cardiovascular health in humans is not established. J Nutr 141:989S–1009S 10.3945/jn.110.131490 21451125

[28] Duthie SJ (2011). Folate and cancer: how DNA damage and DNA repair impact on colon carcinogenesis. J Inherit Metab Dis.

[29] (2011). www.nhs-uk/livewell/5ADAY

[30] Duthie SJ, Ai-guo M, Ross MA (1996). Antioxidant supplementation decreases oxidative DNA damage in human lymphocytes. Cancer Res.

[31] Duthie SJ, Horgan G, De Roos B (2010). Blood folate status and expression of proteins involved in immune function, inflammation and coagulation: biochemical and proteomic changes in the plasma of humans in response to long-term synthetic folic acid supplementation. J Proteome Res.

[32] Duthie GG (1999). Determination of activity of antioxidants in human subjects. Proc Nutr Soc.

[33] Hustad S, Ueland PM, Vollset SE (2000). Riboflavin as a determinant of plasma total homocysteine: effect modification by the methylenetetrahydrofolate reductase C677T polymorphism. Clin Chem.

[34] Singleton VL, Rossi JA (1965). Colorimetry of total phenolics with phosphomolybdic phosphotungstic acid reagents. Am J Enzymol Viticult.

[35] Benzie IF, Strain JJ (1996). The ferric reducing ability of plasma (FRAP) as a measure of “antioxidant power”: the FRAP assay. Anal Biochem.

[36] Rice-Evans CA (2000). Measurement of total antioxidant activity as a marker of antioxidant status in vivo: procedures and limitations. Free Radic Res.

[37] Ou B, Hampsch-Woddill M, Flanagan J (2002). Novel fluorometric assay for hydroxyl radical prevention capacity using fluorescein as the probe. J Agric Food Chem.

[38] Paglia DE, Valentine WN (1967). Studies on the quantitative and qualitative characterization of erythrocyte glutathione peroxidase. J Lab Clin Med.

[39] Aebi H (1984). Catalases in vitro. Methods Enzymol.

[40] Flohé L, Otting F (1984). Superoxide dismutase assays. Methods Enzymol.

[41] Galbraith DA, Watts DC (1980). Changes in some cytoplasmic enzymes from red cells fractionated into age groups by centrifugation in Ficoll/Triosil gradients. Biochem J.

[42] Rutherford L, Reid S (2013) Knowledge, attitudes and motivations to health, 2008–11: a module of the Scottish health survey. NHS Health Scotland, Edinburgh. (http://www.scotpho.org.uk/downloads/scotphoreports/scotpho130424_kam200811.pdf)

[43] Kearney JM, McElhone S (1999). Perceived barriers in trying to eat healthier; results of a pan-EU consumer attitudinal survey. Br J Nutr.

[44] Macdonald HM, Hardcastle AC, Duthie G (2009). Changes in vitamin biomarkers during a 2-year intervention trial involving increased fruit and vegetable consumption by free-living volunteers. Br J Nutr.

[45] Ritchie J, Lewis J (2003) Qualitative research practice: a guide for social science students and researchers. Ist Edition, SAGE Publications Ltd, London

[46] Dittus KL, Hillers VN, Beerman KA (1995). Benefits and barriers to fruit and vegetable intake: relationship between attitudes and consumption. J Nutr Educ.

[47] Casagrande SS, Wang Y, Anderson C (2007). Have Americans increased their fruit and vegetable intake? The trends between 1988 and 2002. Am J Prev Med.

[48] European Food safety Authority (2011) Concise Database summary statistics; total population. http://www.efsa.europe.eu/en/datex/dataexeumenu.htm

[49] http://www.gov.scot/Topics/Health/Healthy-Living/Food-Health/DietaryGoalsScot

[50] Pomerleau J, McKee M, Lobstein T (2003). The burden of disease attributable to nutrition in Europe. Public Health Nutr.

[51] Cox DN, Anderson AS, Reynolds J (1998). Take Five, a nutrition education intervention to increase fruit and vegetable intakes: impact on consumer choice and nutrient intakes. J Nutr.

[52] Chong MF, George TW, Alimbetov D, Jin Y, Weech M, Macready AL, Spencer JPE, Kennedy OB, Minihane A-M, Gordon MH, Lovegrove JA (2013). Impact of the quantity and flavonoid content of fruits and vegetables on markers of intake in adults with an increased risk of cardiovascular disease: the FLAVURS trial. Eur J Nutr.

[53] Paterson E, Gordon MH, Niwat C (2006). Supplementation with fruit and vegetable soups and beverages increases plasma carotenoid concentrations but does not alter markers of oxidative stress or cardiovascular risk factors. J Nutr.

[54] Kolomvotsou AI, Rallidis L, Mountzouris KC, Lekakis J, Koutelidakis A, Efstathiou S, Nana-Anastasiou M, Zampelas A (2013). Adherence to Mediterranean diet and close dietetic supervision increase total dietary antioxidant intake and plasma antioxidant capacity in subjects with abdominal obesity. Eur J Nutr.

[55] Wojcicki JM, Heyman MB (2012). Reducing childhood obesity by eliminating 100% fruit juice. Am J Pub Health.

[56] Papandreou D, Andreou E (2013). Is beverage intake related to overweight and obesity in school children?. Hippokratia.

[57] Moller P, Vogel U, Pedersen A (2003). No effect of 600 grams fruit and vegetables per day on oxidative DNA damage and repair in healthy nonsmokers. Cancer Epidemiol Biomark Prev.

[58] Durga J, Bots ML, Schouten EG (2005). Low concentrations of folate not hyperhomocysteinemia are associated with carotid intima-media thickness. Atherosclerosis.

[59] Bogers RP, Dagnelie PC, Bast A (2007). Effect of increased vegetable and fruit consumption on plasma folate and homocysteine concentrations. Nutrition.

[60] Lettieri-Barbato D, Tomei F, Sancini A, Morabito G, Serafini M (2013). Effect of plant foods and beverages on plasma non-enzymatic antioxidant capacity in human subjects: a meta-analysis. Brit J Nutr.

[61] Dragsted L, Pedersen A (2004). The 6-a-day study: effects of fruit and vegetables on markers of oxidative stress and antioxidant defense in healthy nonsmokers. Am J Clin Nutr.

[62] Brevik A, Karlsen A, Azqueta A, Tirado AE, Blomhoff R, Collins A (2011). Both base excision repair and nucleotide excision repair in humans are influenced by nutritional factors. Cell Biochem Funct.

[63] Tsang C, Higgins S, Duthie GG (2005). The influence of moderate red wine consumption on antioxidant status and indices of oxidative stress associated with coronary heart disease in healthy volunteers. Brit J Nutr.

[64] Duthie SJ, Jenkinson A, Crozier A (2006). Effects of cranberry juice consumption on antioxidant status and biomarkers relating to heart disease and cancer in healthy volunteers. Eur J Nutr.

[65] Duthie GG, Kyle JAM, McE Jenkinson A (2005). Increased salicylate concentrations in urine of human volunteers after consumption of cranberry juice. J Agric Food Sci.

[66] Neville CE, McKinley MC, Draffin CR (2015). Participating in a fruit and vegetable intervention trial improves longer term fruit and vegetable consumption and barriers to fruit and vegetable consumption: a follow-up of the ADIT study. Int J Behav Nutr Phys Act.

[67] Anderson AS, Cox DN, McKellar S (1998). Take Five, a nutrition education intervention to increase fruit and vegetable intakes: impact on attitudes towards dietary change. Brit J Nutr.

[68] Herbert G, Butler L, Kennedy O (2010). Young adults and the 5 A Day Campaign: perceived benefits and barriers to eating more fruits and vegetables. Int J Consum Stud.

