# Supplementary material for: Effect of increasing fruit and vegetable intake by dietary intervention on nutritional biomarkers and attitudes to dietary change: a randomised trial
Source: Eur J Nutr. 2017 May 30;57(5):1855–72. doi: 10.1007/s00394-017-1469-0 (PMC6060837; doi:10.1007/s00394-017-1469-0)
Supplement: Supplementary file 1 — Supplementary material 1 (PDF 23 kb) [file 394_2017_1469_MOESM1_ESM.pdf]

Intervention	Day 1	Day 2	Day 3	Day 4	Day 5	Day 6	Day 7
Fruit	strawberries	apple	plums	apple	plums	grapes	strawberries
	orange	kiwi fruit	pear	grapes	orange	pear	banana
Salad vegetables	mixed leaf	mixed leaf	mixed leaf	mixed leaf	mixed leaf	mixed leaf	mixed leaf
		tomatoes	tomatoes	tomatoes	tomatoes	tomatoes	tomatoes
		red pepper	cucumber		red pepper	cucumber	
Vegetable	crunchy medley	onion, sweet pepper & mangetout	Winter selection	Winter selection	onion, sweet pepper & mangetout	mixed cabbage	carrot, cauliflower & broccoli mix
	carrots					carrots	green beans
Fruit Juice	OJ	OJ	OJ	OJ	OJ	OJ	OJ
	AJ	AJ	AJ	AJ	AJ	AJ	AJ
Daily weight (g)	480	480	480	480	480	480	480
Juice (ml)	300	300	300	300	300	300	300

Supplementary Table 1. Fruit, vegetable and juice provision.

Participants in the intervention group were asked to consume all of the fruits, vegetables and 2 small glasses (150ml) of fruit juices (apple; AJ or orange; OJ) from the foods in the table, in addition to their normal intake, ensuring equivalent consumption of fruits and vegetables. All fruit, vegetables and juice were provided (twice weekly) to the participants. The same fruits and vegetables were provided to all volunteers unless a participant declared a strong dislike for a particular item, in which case it was replaced. Crunchy medley (cabbage mixture); Winter selection (carrots, cauliflower, sprouts); Mixed leaf (rocket, mizuria, red chard).
